# Management of Parkinson's Disease During Pregnancy: Literature Review and Multidisciplinary Input

**DOI:** 10.1002/mdc3.12925

**Published:** 2020-04-09

**Authors:** Caitlin Young, Rhiannon Phillips, Louise Ebenezer, Rodi Zutt, Kathryn J. Peall

**Affiliations:** ^1^ Cardiff University School of Medicine, Cardiff University Cardiff United Kingdom; ^2^ Division of Population Medicine, School of Medicine Cardiff University Cardiff United Kingdom; ^3^ Princess of Wales Hospital Abertawe Bro Morgannwg University Health Board Bridgend Wales United Kingdom; ^4^ Department of Neurology Haga Teaching Hospital The Hague The Netherlands; ^5^ Neuroscience and Mental Health Research Institute, Cardiff University Cardiff United Kingdom

**Keywords:** movement disorders, Parkinson's disease

## Abstract

**Background:**

There are no standardized clinical guidelines for the management of Parkinson's disease (PD) during pregnancy. Increasing maternal age would suggest that the incidence of pregnancy in women diagnosed with PD is likely to increase.

**Objective:**

To evaluate the evidence for the treatment of PD during pregnancy and to canvass opinion from patients and clinical teams as to the optimum clinical management in this setting.

**Methods:**

This involved (1) a literature review of available evidence for the use of oral medical therapy for the management of PD during pregnancy and (2) an anonymized survey of patients and clinical teams relating to previous clinical experiences.

**Results:**

A literature review identified 31 publications (148 pregnancies, 49 PD, 2 parkinsonism, 21 dopa‐responsive dystonia, 32 restless leg syndrome, 1 schizophrenia, and 43 unknown indication) detailing treatment with levodopa, and 12 publications with dopamine agonists. Adverse outcomes included seizures and congenital malformations. Survey participation included patients (n = 7), neurologists (n = 35), PD nurse specialists (n = 50), obstetricians (n = 15), and midwives (n = 20) and identified a further 34 cases of pregnancy in women with PD. Common themes for suggested management included optimization of motor symptoms, preference for levodopa monotherapy, and normal delivery unless indicated by obstetric causes.

**Conclusions:**

This study demonstrates the paucity of evidence for decision‐making in the medical management of PD during pregnancy. Collaboration is needed to develop a prospective registry, with longitudinal maternal and child health outcome measures to facilitate consensus management guidelines.

Approximately 5% of Parkinson's disease (PD) diagnoses are made in individuals younger than 40 years, meaning that women with early‐onset PD may become pregnant after diagnosis.^1^ The incidence of pregnancy in women with PD is unclear, although it is likely to rise given the trend toward increasing maternal age and that no data to date indicate a reduction in fertility for those women diagnosed with PD.^2^ Previous case series have documented fetal and maternal outcomes in multiple pregnancies, including adverse events such as spontaneous abortion. However, information about the use and safety of medication for the treatment of PD during pregnancy is largely anecdotal and lacks long‐term follow‐up maternal and child outcome data. The lack of evidence‐based practice and standardized clinical guidelines means that clinicians and women with PD face uncertainty as to how best to plan care during this period. This study seeks to evaluate and summarize currently available evidence for the management of PD during pregnancy and to determine the experiences of both patients and clinical teams in this clinical setting.

## Methods

This study includes (1) a structured literature review of available evidence relating to the use of medication and deep brain stimulation (DBS) to treat PD during pregnancy and (2) a survey of patient and clinical team experiences of PD management during pregnancy.

### Literature Search

Our literature review aimed to address the obstetric outcomes when medication and/or DBS were used to treat PD during pregnancy and to assess the quality of evidence against GRADE criteria (grading of Recommendations, Assessments, Development and Evaluation). Reports meeting the following criteria were eligible for inclusion: (1) those relating to the use of levodopa, dopamine agonist, monoamine oxidase‐B (MAO) inhibitors, or catechol‐O‐methyl‐transferase (COMT) inhibitors or antimuscarinic therapy in pregnant women, irrespective of diagnosis, as well as DBS for those with a diagnosis of PD; (2) an English‐language abstract; (3) data and observations from pregnancy in humans rather than other mammalian species. To maximize reach, data relating to the use of PD therapies in other dopamine‐responsive conditions such as restless leg syndrome and dopa‐responsive dystonia were also included. No restriction was placed on the date of publication, with information sourced using the MEDLINE and Web of Science databases. Additional articles were also identified from the reference list of screened articles. The database search strategy is summarized in Supplementary Figure S1. Those articles included were subsequently divided into case reports, small case series (n < 5), large case series (n > 5), and larger observational studies (Supplementary Table S1). The information collated included name or class of dopaminergic medication, number of pregnancies exposed, reason for treatment (maternal diagnosis), and pregnancy outcome. The GRADE criteria were used to assess the quality of evidence relating to each treatment with the summary measure determined by the total number of live births, spontaneous abortions, terminations of pregnancy, and still births with each form of therapy.

### Survey Data Collection

Via an online survey, data were collected on the following 5 key domains: (1) medication to treat PD symptoms, (2) PD symptoms during pregnancy, (3) organization of clinical care, (4) adverse obstetric events and delivery, (5) postpartum period. Informed consent was obtained from 5 groups: individuals diagnosed with PD who had been pregnant since diagnosis, neurologists, obstetricians, midwives, and PD specialist nurses. The organizations involved in contacting these groups are summarized in Supplementary Figure S2. Health care professionals without previous clinical experience in this setting were also invited to share suggested management plans to gain a wider context of opinion.

### Data Analysis

Nominal and multiple‐choice survey responses were analyzed descriptively. Open‐text responses were coded according to content, and an inductive, data‐driven coding approach employed. Content analysis identified key themes, and constant comparison enabled a search for emerging themes.

## Results

### Literature Review: Clinical Evidence for the Use of Antiparkinsonian Medication During Pregnancy

Supplementary Table S1 and Table 1 summarize the publications reviewed and outcomes, respectively.^3^, ^4^, ^5^, ^6^, ^7^, ^8^, ^9^, ^10^, ^11^, ^12^, ^13^, ^14^, ^15^, ^16^, ^17^, ^18^, ^19^, ^20^, ^21^, ^22^, ^23^, ^24^, ^25^, ^26^, ^27^, ^28^, ^29^, ^30^, ^31^, ^32^, ^33^, ^34^, ^35^, ^36^, ^37^, ^38^, ^39^, ^40^, ^42^ In brief, 31 publications reported the use of levodopa in 148 pregnancies, with examples of reported adverse outcomes including congenital malformation (n = 8) and seizures.^4^, ^5^, ^6^, ^7^ Two publications provided results of genetic testing, including a total of 4 cases with *Parkin* mutations.^21^, ^22^ Of the 109 levodopa‐exposed pregnancies for which outcomes were available, 83% resulted in live births (n = 91), 8% were electively terminated (n = 9), and 9% resulted in spontaneous abortion (n = 10). Fewer publications included the use of dopamine agonists (n = 12), antimuscarinic medication (n = 4), COMT inhibitors (n = 4), monoamine‐oxidase B inhibitors (n = 3), and DBS (n = 4). The largest case series of DBS during pregnancy identified 11 individuals with 18 births (PD = 3, dystonia = 5, Tourette's syndrome = 2, obsessive‐compulsive disorder = 1). Of the 3 cases diagnosed with PD, 1 stopped her medication during pregnancy and resumed at the same dose postpartum, another changed from a dopamine agonist to levodopa and back to a dopamine agonist postpartum, and the third continued her treatment of a dopamine agonist and MAO inhibitor throughout.^21^ None of these women breastfed in the postpartum period owing to concerns of the impact of their oral medical therapy. The quality of evidence is summarized according to the GRADE criteria (Table 2).

**Table 1:** Outcomes following in utero exposure to Parkinson's medications in the treatment of neuro‐psychiatric disorders

In Utero Exposure to Parkinson's Medication						
No. Pregnancies	Indication	Drug	Range Maximum Dose (mg/day)	Duration Exposure (Weeks)	Pregnancy Outcome	Complications
**In utero exposure to levodopa**						
*Case reports: 22 publications*						
32 pregnancies	26 PD, 2 P, 4 DRD	Levodopa preparations	100–1500 No data (12)	6–40 No data (3)	31 live births (4 prem), 2 SA	Neonate seizure 1‐hour postpartum, Placental abruption, VSD in 1 twin, PPROM in 2 pregnancies
			
*Small case series: 3 publications*						
12 pregnancies	9 PD, 3 DRD	Levodopa preparations	1250–4000	36 No data (9)	10 live births, 2 SA	
*Large case series and observational studies: 6 publications*						
104 pregnancies	14 PD, 14 DRD, 32 RLS, 1 psych, no data (43)		100–400 No data (52)	12–36 No data (52)	50 live births (8 prem), 6 SA, 9 TOP, 2 LTF, No data (37)	3 minor anomalies (PFO + PDA, talipes varus, nasal deformity), 1 preeclampsia 1 premature infant developed fetal distress during labor, eventually resolved
Total pregnancies: 148			91 live births, 10 SA, 9 TOPs, 2 LTF, 37 no data			
**In utero exposure to dopamine agonists**						
*Case reports: 9 studies*						
10 pregnancies	10 PD	PRAM (3)	0.75–4.5	29–36+	11 live births (4 prem)	1 placental abruption, VSD in 1 twin, 1 neonate seizure 1‐hour postpartum
		PER (1)	3			
		CAB (2)	1–4			
		ROP (2)	1.5–1.88			
		BROM (2)	20–25			
*Large case series and observational studies: 3 studies*						
151 pregnancies	1 DRD, 20 RLS, 13 PD, No data (117)	BROM (20)	No data	3–36+	4 SA, 1 TOP, 31 live births (6 prem) including 2 pairs of twins 1 subsequent neonatal death due to liver enzyme deficiency	1 neonatal death 1 prem infant developed fetal distress during labor, eventually resolved 1 small for gestational age
		PRAM (84)	1.125–4.5			
		CAB (31), ROP (10)	No data			
		ROT (2)	8–6			
		APOM (1)	No data			
		PIRI (3)	No data			
			100–300			
Total pregnancies: 161			42 live births (including 3 pairs of twins) and 1 subsequent neonatal death, 4 SA, 1 TOP, 117 no data			
**In utero exposure to antimuscarinics**						
*Case reports: 4 publications*						
7	2 dystonia, 4 SCZ, 1 PD	TRI	2‐50	36‐42	6 healthy neonates, 1 SA	
**In utero exposure to COMT inhibitors**						
*Case reports: 4 publications*						
4	4 PD	ENTA	200‐700	12‐36	5 live births (including twins)	1 neonate seizure 1‐hour postpartum PPROM in twin pregnancy with EMCS at 35 weeks Small VSD in 1 twin
**In utero exposure to MAO inhibitors**						
*Case reports: 2 publications*						
2	2 PD	SELE	7.5, 10	29‐40	3 live births (including twins)	PPROM at 35 weeks with EMCS Small VSD in 1 twin
*Large case series: 1 publication*						
7	7 PD	RASA	1	4‐36+	7 live births (2 prem), 1 neonatal death	1 neonatal death of a twin due to liver enzyme deficiency
**In utero exposure to DBS**						
*Case reports: 1 publication*						
1	Dystonia			Used throughout gestation	1 live birth	
*Small case series: 1 publication*						
4	Dystonia			Used throughout gestation	4 live births	Intrauterine growth retardation in 1 pregnancy
*Large case series: 2 publications*						
18	PD, dystonia, TS, OCD			Used throughout gestation	18 live births (including twins), 1 SA	

36+ denotes levodopa exposure for full duration of pregnancy with term delivery, where exact gestational age at delivery is unavailable.

COMT, catechol‐O‐methyl‐transferase; MAO, monoamineoxidase‐B; DBS, deep brain stimulation; PD, Parkinson's disease; P, parkinsonism; DRD, dopa‐responsive dystonia; RLS, restless leg syndrome; SCZ, schizophrenia; TS, Tourette's syndrome; OCD, obsessive‐compulsive disorder; SA, spontaneous abortion; TOP, termination of pregnancy; LTF, lost to follow‐up; PRAM, pramipexole; PER, pergolide; CAB, cabergoline; ROP, ropinirole; BROM, bromocriptine; ROT, rotigotine; APOM, apomorphine; PIRI, piribedil; TRI, trihexyphenidyl; ENTA, entacapone; SELE, selegiline; RASA, rasagiline; prem, premature; VSD, ventricular septal defect; PPROM, preterm premature rupture of membranes; PFO, patent foramen ovale; PDA, patent ductus arteriosus; EMCS, emergency caesarean section.

**Table 2:** GRADE quality of evidence

Drug	No. of Studies/Pregnancies	Design	Quality	Consistency	Directness	Overall Quality
Levodopa preparation	31 studies, 148 pregnancies	Case reports: 22 (32 pregnancies) Small case series: 3 (12 pregnancies) Large case series: 4 (25 pregnancies) Observational studies: 2 (80 pregnancies)	Predominantly case studies or case series; limited generalizability Observational studies are based on data from drug registries; no control group for comparison Many studies lack data regarding drug dose and duration	Case report/series have largely consistent positive outcomes. No major fetal abnormalities reported with levodopa use	Outcome reasonably measure direct; fetal health and absence of malformation ∼ safety of levodopa in pregnancy > 51/143 patients treated for conditions other than PD—likely require lower doses of levodopa Limited infant follow‐up—adverse effects may not become apparent until later life	Low—further research is likely to have an important impact on confidence in the safety of levodopa in pregnancy
Dopamine agonists	12 studies, 161 pregnancies	Case reports/small case series: 9 (10 pregnancies) Large case series/observation studies: 3 (151 pregnancies)	Case reports provide limited generalizability, cannot comment on causality	Case reports have largely consistent positive outcomes. No major fetal abnormalities reported	Outcome measure direct; fetal health and absence of malformation ∼ safety of DA in pregnancy Limited infant follow‐up—adverse effects may not become apparent until later life	Very low—any estimate of safety is very uncertain
Antimuscarinics	4 studies, 7 pregnancies	Case reports/small case series: 4 (7 pregnancies)	Limited generalizability, cannot comment on causality	Too few studies to comment on consistency	Outcome measure direct; fetal health and absence of malformation ∼ safety of antimuscarinics in pregnancy Limited infant follow‐up—adverse effects may not become apparent until later life	Very low—any estimate of safety is very uncertain
COMT inhibitors	4 studies, 4 pregnancies	Case reports/small case series: 4 (4 pregnancies)	Limited generalizability, cannot comment on causality	Too few studies to comment on consistency	Outcome measure direct; fetal health and absence of malformation ∼ safety of COMT inhibitors in pregnancy Limited infant follow‐up—adverse effects may not become apparent until later life	Very low—any estimate of safety is very uncertain
MAO inhibitors	3 studies, 9 pregnancies	Case reports/small case series: 2 (2 pregnancies) Large case series: 1 (7 pregnancies)	Limited generalizability, cannot comment on causality	Too few studies to comment on consistency	Outcome measure direct; fetal health and absence of malformation ∼ safety of COMT inhibitors in pregnancy Limited infant follow‐up—adverse effects may not become apparent until later life	Very low—any estimate of safety is very uncertain
DBS	4 studies, 23 pregnancies	Case reports/small case series: 2 (5 pregnancies) Large case series: 2 (18 pregnancies)	Limited generalizability, cannot comment on causality	Too few studies to comment on consistency	Outcome measure direct; fetal health and absence of malformation ∼safety of DBS in pregnancy Limited infant follow‐up—adverse effects may not become apparent until later life	Very low‐ any estimate of safety is very uncertain

GRADE, Grading of Recommendations, Assessment, Development and Evaluation; COMT, catechol‐O‐methyl‐transferase; MAO, monoamine‐oxidase; DBS, deep brain stimulation; PD, Parkinson's disease; DA, dopamine agonist.

### Survey Outcomes: Patients, Neurologists, PD Nurse Specialists, Obstetricians, and Midwives

Our survey identified 34 pregnancies in women with PD, with medication continued in 15, and 2 reported complications (Table 2).

#### Women Diagnosed with PD with Subsequent Pregnancy

A total of 7 women completed our survey regarding 10 pregnancies, resulting in 8 healthy live births, 1 stillbirth (24 weeks), and 1 pregnancy with unknown outcome (Table 2). Three women were diagnosed with PD during pregnancy (20–48 years), and 3 women received oral medical therapy during 4 pregnancies. One patient reported an improvement in motor symptoms despite withdrawal of all PD medications during this period. Two patients (2 and 6) required oral medical therapy postpartum due to worsening motor symptoms.

#### Neurologists

A total of 35 neurologists responded to our survey, 8 of whom had experience caring for women with PD during 12 pregnancies (Table 3 and Supplementary Table S2). Management suggestions included reviewing medication safety and using as few medications as possible, particularly preconception and during the first trimester. Emphasis was placed on maintaining good motor symptom management during pregnancy, and if required oral levodopa monotherapy was preferred. They also suggested regular review, referral to specialist movement disorder clinics during the antepartum period, and close working with other members of the multidisciplinary team.

**Table 3:** Neurologist, PD nurse specialist, obstetrician, and patient experiences of PD during pregnancy

				Parkinson’s medication in pregnancy							
**Case No.**	**No of cases**	**Aware of plans to conceive**	**Informed of pregnancy (trimester)**	**Medication during pregnancy**	**Medication changes during pregnancy**	**Post‐partum medication change**	**PD symptoms during pregnancy a) Motor b) Non‐motor**	**PD symptoms post‐partum a) Motor b) Non‐motor**	**Obstetric outcomes**	**Antenatal organisation and provision of care a) Communication between obstetrics & neurology b) Joint obstetric‐neurology review**	**Post‐partum organisation and provision of care a) Neurology informed of birth b) Inpatient neurology review**
**A. Neurologists**											
**1**	1	N	‐	‐	No change	‐	a) No change b) No change	a) No change b) No change	Live birth	a) Yes b) No	a) Yes b) Yes
**2**	1	N	1^st^		No change		a) No change b) No change	a) No change b) No change	Live birth	a) Yes b) Yes	a) N/A b) N/A
**3**	5	Y	1^st^	Levodopa alone (3) Withheld all medications (2)	Levodopa increased (1)	Return to usual regimen	a) Generally worse b) 1 patient became depressed	a) No change b) Mood changes	Live births	a) Yes b) Yes (2 cases), No (3 cases)	a) Yes b) Yes (2 cases)
**4**	1	Y	1^st^		No change	No change	a) No change b) No change	a) No change b) No change	Live birth (twins)	a) Yes b) No	a) No b) No
**5**	1	N	1^st^	‐	No change	No change	a) No change b) No change	a) N/A b) N/A	TOP	a) No b) No	a) N/A b) N/A
**6**	1	N	1^st^	Stalevo 300‐400mg /day	No change	Stalevo 400‐500mg/ day	a) Bradykinesia worse b) No change	a) Bradykinesia improved b) No change	Live birth	a) Yes b) No	a) No b) No
**7**	1	N	1^st^	Levodopa	Initially stopped, re‐started.	No change	a) Worse without medication b) No change	a) No change b) No change	Live birth	a) Yes b) No	a) No b) No
**8**	1	N	1^st^	‐	No change	No change	a) No change b) Psychiatric symptoms	a) No change b) No change	Live birth	a) Yes b) No	a) No b) No
**B. PD Nurse Specialists**											
**1**	1	Y	1st	All stopped except Sinemet 6.25mg tds	No change	N/A	a) No change b) No change	a) N/A b) N/A	SA 12 weeks	a) Yes b) ‐	a) – b) N/A
**2**	2	N	2nd	Madopar 100/25 tds	No change	No change	a) No change b) No change	a) No change b) No change	Live birth	a) Yes b) Yes	a) Yes b) No
**3**	2	Y + N	1st in both	1 stopped meds pre‐conception, 1 reduced pramipexole.	No change	Return to usual regimen	a) No change b) No change	a) Increased bradykinesia and off symptoms b) Anxiety, poor sleep	Live births	a) Yes b) Joint review with midwife. Found midwife disinterested in birth plan.	a) No b) No
**4**	1 (2P)	N	2^nd^	Levodopa. Cabergoline withheld.	Levodopa increased.	Cabergoline restarted (6 weeks).	a) No change b) No change	a) No change b) No change	Live births	a) Yes b) No	a) Yes b) Yes
**5**	1	N	1st	Dopamine agonist withheld.	No change.	Nil	a) Bradykinesia, b) Fatigue.	a) No change b) No change	Live birth	a) Yes b) No	a) No b) No
**C. Obstetricians**											
**Case No.**	**No of cases**	**Stage of first involvement**	**Frequency of obstetric review**	**Change to regular schedule of antenatal obstetric care**	**Joint obstetric‐neurology antenatal review** **a) Joint review undertaken** **b) Would joint review be helpful?**			**Obstetric outcome**	**Immediate post‐partum care** **a) Inpatient neuro review** **b) Breastfeeding** **c) Length of hospital stay**		**Post‐partum outpatient obstetric review**
**1**	2	Pre‐conception 1^st^	3‐4 times	More frequent antenatal clinic review	a) Yes, b) Yes; ‘spare the women additional trips to hospital’			Live births, VD. No complications	a)No, b)Unable, c) <3 days		a) No outpatient obstetric review
**2**	2	2^nd^ trimester	Twice	Increased visits, serial growth monitoring	a) No, b) Yes; *‘allow discussion between obstetrics and neurology’*			Live births, VD. No complications	a) Yes, b) – c) 4 days		a) No outpatient obstetric review

**D. Women diagnosed with PD**						
**Case No.**	**Patient demographic**	**Pregnancy management** **a) Medication, b) PD symptoms during pregnancy**	**Obstetric outcomes**	**Pregnancy care** **a) Provision of antenatal care** **b) Frequency of antenatal neurology review**	**Support and information** **a) Level of support from healthcare team, b) Provision of information during pregnancy**	**Post‐partum** **a) Breastfeeding, b) Attendance at mother and baby groups, c) Attendance at PD support groups, d) Medication change post‐partum**
**1**	Diagnosed at 20 years, G1P1, FH ‐	a) No change, Levodopa/carbidopa during pregnancy b) No change.	Live birth. EMCS, 37 weeks.	a) Consultant‐led, b) No change	a) Well‐supported, b) Inadequate	a) Breastfed for 8 weeks, b) Attended , c) Did not attend, d) No change
**2**	Diagnosed at 29 years, G2P2, FH +	a) No change. Cannot recall for 1^st^ pregnancy. Requip XL in 2^nd^ pregnancy, b) General worsening of motor symptoms.	Live birth. Live birth. VD, 36 weeks.	a) Consultant‐led, b) No change (6 monthly)	a) Well‐supported, b) Adequate	a) Not breastfed, b) Attended, c) Did not attend, d) Dose increased
**3**	Diagnosed at 25 years, G2P?^a^, FH +	a) Sinemet, Rasagiline, Ropinirole withheld. No medications taken. b) Improvement in motor symptoms, mood and energy. Most noticeable in 2^nd^ trimester.	Live birth. VD, 9 weeks. Admitted week 38. Neonatal hypoglycaemia post‐partum; stayed 1 week in hospital.	a) Consultant‐led, b) No change (seen once during pregnancy)	a) Poorly supported, b) Inadequate	a) Breastfed for 1 week. Stopped to re‐start medication, b) Attended, c) Did not attend
**4**	Diagnosed at 44 years, G1P1, FH ‐	a) None taken‐ no regular medication, b) PD presented during pregnancy and worsened in 2^nd^ trimester.	Live birth. EMCS, 35 weeks. HELLP syndrome. Infant in NICU for 24 hrs.	a) Midwife‐led, b) No formal diagnosis until post‐partum	a) Well‐supported, b) Inadequate	a) Not breastfed, b) Attended, c) Did not attend
**5**	Diagnosed at 26 years, G2P1, FH ‐	a) Cabergoline and orphenadrine withheld. Madopar 50/12.5mg during pregnancy b) Increased tremor.	Stillbirth, 24 weeks Live birth. AD, full term	a) Midwife‐led, b) More frequent (seen 3 times)	a) Unsure, b) Inadequate	a) Not breastfed, b) Did not attend, c) Did not attend, d) Resumed medication
**6**	Diagnosed at 33 G1P1, FH ‐	a) No regular medication and none during pregnancy. PD diagnosed in early pregnancy. b) Worsening bradykinesia, UL tremor, dexterity, sialorrhoea. Most marked 3^rd^ trimester	Live birth. VD, 41 weeks. Maternal pyrexia post‐partum; given IV antibiotics overnight.	a) Consultant‐led b) First neurology consultation at 14 weeks; seen several times thereafter	a) Well‐supported b) Unsure	a) Not breastfed. b) Attended c) Attended d) Sinemet initiated
**7**	PD Diagnosed at 48 G1P1, FH ‐	a) No regular medication. PD diagnosed post‐partum. b) Worsening; tremor in 1st and 3rd trimesters. Micrographia.	Live birth. AD, 40 weeks. Neonatal respiratory difficulties‐ infant in NICU for 36 hrs.	a) Consultant‐led b) First neurology consultation at 12 weeks; no regular neurology review during pregnancy. Formal diagnosis post‐partum	a) Well‐supported. b) Inadequate	a) Breastfed for 8 months. Stopped to start medication b) Attended c) Did not attend

a Outcome of second pregnancy not specified by respondent.

PD, Parkinson's disease; N/A, not applicable; tds, three times daily; SA, spontaneous abortion; 2P, two pregnancies; VD, vaginal delivery; G, gravidity; P, parity (eg, G1P1 = gravida 1, para 1); FH −, family history; FH +, family history; UL, upper limb; EMCS, emergency caesarean section; HELLP, haemolysis, elevated liver enzymes, low platelets syndrome; NICU, neonatal intensive care unit; AD, assisted delivery.

#### PD Nurse Specialists

A total of 50 responses were obtained, 5 of whom had experience of caring for patients with PD during 8 pregnancies (Table 3 and Supplementary Table S2). Suggestions focused on the antenatal period, including reviewing medication and aiming to minimize oral medical therapy. There was wide support for adopting a multidisciplinary approach (n = 19). Suggestions for organization of care included open access to neurology services (n = 1) and more frequent monitoring (n = 6).

#### Obstetricians

Fifteen responses were obtained from obstetricians, with 2 having had experience managing 4 pregnancies in mothers with PD (Table 3 and Supplementary Table S2). Twelve suggested obstetric led care due to the unknown medication risk in pregnancy and the potential for worsening motor symptoms. There was consensus that a normal schedule of antenatal appointments should be followed with increased review if problems arose. Additional recommendations included prepregnancy counseling, monthly joint clinics with the neurology team, and coordination of antenatal appointments with fetal growth scans. None advised delivery by caesarean section, with this being reserved for obstetric indications only. Suggestions for postpartum management included standard care, inpatient neurology review within 24 hours of delivery, and obstetric high‐dependency monitoring.

#### Midwives

A total of 20 midwives responded to the survey, none of whom had experience of caring for women with PD during pregnancy. Seventeen shared suggestions for pregnancy care (Supplementary Table S2. Antenatally, these included the following: obstetric‐led care (n = 7), multidisciplinary team approach (n = 8), involvement of physiotherapists to aid balance difficulties (n = 4), and the offer of home visits to avoid long waits in antenatal clinics (n = 2). Active, mobile labor was advised, although the potential for women to tire, guided by their experience of other chronic disorders, was highlighted alongside the midwifery preference for delivery in an obstetric unit or midwife‐led unit alongside an obstetric center.

## Discussion

This study represents the first to synthesize evidence relating to clinical outcomes of the management of PD during pregnancy and investigate care experiences from patient and multidisciplinary team perspectives.

### Medication During Pregnancy

#### Literature Review

Our literature review demonstrates the paucity of evidence for the safety of dopaminergic therapy during pregnancy, with levodopa the preferred form of treatment. Ten pregnancies, from a total of 148, resulted in spontaneous abortion (9.2%), and 3 live births were associated with fetal congenital abnormalities including patent foramen ovale and ductus arteriosus.^3^ The rates of clinically recognized pregnancies resulting in fetal loss in the general population are estimated to be 10% to 24%, indicating no excess rate among this patient group, particularly in the context of exposure to levodopa therapy.^41^ Fewer studies related to the use of dopamine agonists, antimuscarinic drugs, and COMT and MOA‐B inhibitors during pregnancy, and therefore estimates of fetal loss are more difficult to determine. Spontaneous abortion was reported in 4 cases of pramipexole monotherapy.^3^, ^42^

Our literature search also included data relating to the use of medication in the treatment of other dopamine‐responsive disorders during pregnancy such as restless legs syndrome and dopa‐responsive dystonia. The underlying aetiology of these disorders is distinct from that of PD and may independently impact pregnancy irrespective of medication. There was substantial variation in the dose of all prescribed medication (Table 1) and understanding of the risk of obstetric complications is limited by the majority of evidence provided in the form of case reports. Furthermore, PD medications were frequently coprescribed, making it difficult to elucidate the effects of individual drugs. Four publications relating to the use of DBS during 23 pregnancies were also identified (Supplementary Table S1). All operations were undertaken prepregnancy with 23 live births and 1 spontaneous abortion in the first few weeks of pregnancy reported. No complications with the use of DBS during pregnancy were reported.

#### Multidisciplinary Survey Outcomes

Our survey found 88.2% (n = 30/34) of the identified pregnancies resulted in a live birth, and 5.9% (n = 2/34) ended in spontaneous abortion, below the estimated rates in the general population.^41^ Where medication was continued, there was a preference for levodopa. However, these results are retrospectively reported, and due to recruitment methods, potentially not representative of the spectrum of women diagnosed with PD who have subsequently become pregnant.

### Parkinson's Symptoms During Pregnancy

Published literature to date suggests that women experience variation in their PD symptoms during pregnancy, with early reports suggesting that 65% of women experienced worsening of their symptoms despite the continuation of medical therapy.^12^ The physiological mechanisms by which pregnancy can result in symptomatic change is poorly understood. Altered pharmacokinetics due to the expansion in plasma volume may reduce peak serum concentrations of oral medical therapy, whereas changes to gastrointestinal absorption and increases in estimated glomerular filtration rate (eGFR) may affect the availability and renal elimination of drugs.^43^ In keeping with this, our survey identified variation in the evolution of motor symptoms during the course of pregnancy, although this may have related to a number of factors, including ongoing adjustments to the dose of medical therapy for which no serum measurements were available.^1^

Half of the women surveyed noted worsening of symptoms during pregnancy, 13 reported no change, and 1 patient reported an overall improvement in motor symptoms, mood, and energy levels during 2 pregnancies. Where symptoms worsened, 60% (n = 9) did so after all or adjuvant medications were withheld or doses reduced, whereas 25% (n = 4) noted symptom worsening while receiving treatment with levodopa monotherapy. Only 15% (n = 2) of women whose symptoms worsened did so despite no change to PD medications. These reports suggest that although PD symptoms during pregnancy are likely to vary between individuals, the maintenance of at least prepregnancy treatment levels is likely to limit symptomatic fluctuation.

### Organization of Care

Studies of other chronic disorders (eg, rheumatoid arthritis) in pregnancy emphasize the need for well‐coordinated multidisciplinary involvement, with decision aids demonstrating enhanced shared decision‐making.^44^ Despite this, there are no currently available guidelines on obstetric best practice in the management of PD during pregnancy, and only 2 patients in this cohort received more frequent antenatal neurology input. All of the obstetricians consulted felt that antenatal care should be consultant led and follow a normal schedule of antenatal appointments. Although joint obstetric/neurology review was only undertaken in 3 cases in this study, both clinician groups advocated enhanced communication between teams.

### Adverse Events and Delivery

To date, there is no evidence to suggest higher rates of fetal or maternal complications, fertility difficulties, or birth‐related complications in women with PD.^1^ Obstetrician responses in this survey felt there was no indication to alter the standard of postpartum care (4/15 [27%]) and that a diagnosis of PD would not contraindicate vaginal delivery, suggesting that delivery by caesarean section should be reserved for obstetric indications only. Information relating to the mode of delivery was available for 12 pregnancies: 8 vaginal deliveries, 2 emergency caesarean sections (17%), and 2 assisted deliveries (17%). The rate of emergency caesarean section is ~15% in the United Kingdom, broadly comparable with that observed in our data set.^45^

### Postpartum Period, Breastfeeding, and Support

The challenges facing new mothers with PD are poorly understood, with deteriorating fine motor skills often presenting functional difficulties in undertaking daily tasks. Decisions relating to breastfeeding are complicated by limited information regarding the potential risk of medication to infants, although plasma and breastmilk levodopa concentrations in a single study estimated the level of exposure to be low (0.016–0.023 mg/kg/day).^24^ The inhibitory effects of levodopa and dopamine agonists on prolactin synthesis suggests they may suppress lactation, although 2 women in this cohort were able to breastfeed for a limited time.

## Conclusion

This study has collated information from a number of distinct sources, highlighting several key aspects. The majority of outcome data for pregnancies of women diagnosed with PD are linked with use of levodopa treatment during this period, with outcome data only available in a small number of cases for those treated with other forms of dopaminergic therapy. The results from our systematic review indicate no excess rates of miscarriage, stillbirth, or congenital deformity among this patient group compared with the general population. Patient and multi‐disciplinary team (MDT) survey responses suggest that an optimized care plan would include close cooperation between neurology and obstetric teams during pregnancy and delivery. However, the most important element highlighted is the need for an international prospective registry for women diagnosed with PD during and after pregnancy, similar to those for other chronic neurological disorders. A registry would aid in the development of consensus guidelines for clinical care in this setting and provide longer term follow‐up data on infant and childhood development to better aid therapeutic decision‐making.

## Author Roles

(1) Research Project: A. Conception, B. Organization, C. Execution; (2) Statistical Analysis: A. Design, B. Execution, C. Review and Critique; (3) Manuscript Preparation: A. Writing of the First Draft, B. Review and Critique.

C.Y.: 1A, 1B, 1C, 2A, 2B, 2C, 3A, 3B

R.P.: AB, 2C, 3B

L.E.: AB, 2C, 3B

R.Z.: AB, 2C, 3B

K.J.P.: 1A, 1B, 1C, 2A, 2B, 2C, 3A, 3B

## Disclosures

### Ethical Compliance Statement

Ethical approval for this study was provided by Cardiff University School of Medicine Research Ethics Committee (reference: 18/05). Informed consent was obtained from all participants in this study, including both patients and clinical team members. We confirm that we have read the Journal's position on issues involved in ethical publication and affirm that this work is consistent with those guidelines.

### Funding Sources and Conflicts of Interest

K.J.P. is funded by a Medical Research Council Clinician‐Scientist Fellowship (MR/P008593/1) and grants from the Dystonia Medical Research Foundation, The Dystonia Society, Fight for Sight, and the Jacques and Gloria Gossweiler Foundation. The authors report no conflicts of interest in relation to this work.

### Financial Disclosures for Previous 12 Months

The authors report no financial disclosures for the previous 12 months.

## Supporting information


**Supplementary Figure S1.** Schematic representation of the search terms used during the systematic literature review. Blue boxes represent the research terms used and number of publications identified. Green boxes represent additional publications identified, and orange boxes are those excluded as they were not considered relevant to this review. Articles were divided into case reports (n = 1), smaller case series (n < 5), and larger case series or cohort studies (n > 5). COMT inhibitor, catechol‐O‐methyl‐transferase inhibitor; MAOI, monoamine‐oxidase inhibitors.

**Supplementary Figure S2.** Schematic representation or sources of participant recruitment from clinical and patient sectors (green, patient recruitment; orange, neurology recruitment; blue, Parkinson's disease nurse specialist recruitment; yellow, midwifery recruitment; purple, obstetric recruitment).

**Supplementary Table S1.** Details of publications identified in systematic literature review.
**Supplementary Table S2.** Summary of questionnaire free‐text responses.
