# Supplementary figures and images for: Management of Parkinson's Disease During Pregnancy: Literature Review and Multidisciplinary Input
Source: Mov Disord Clin Pract. 2020 Apr 9;7(4):419–30. doi: 10.1002/mdc3.12925 (PMC7197310; doi:10.1002/mdc3.12925)

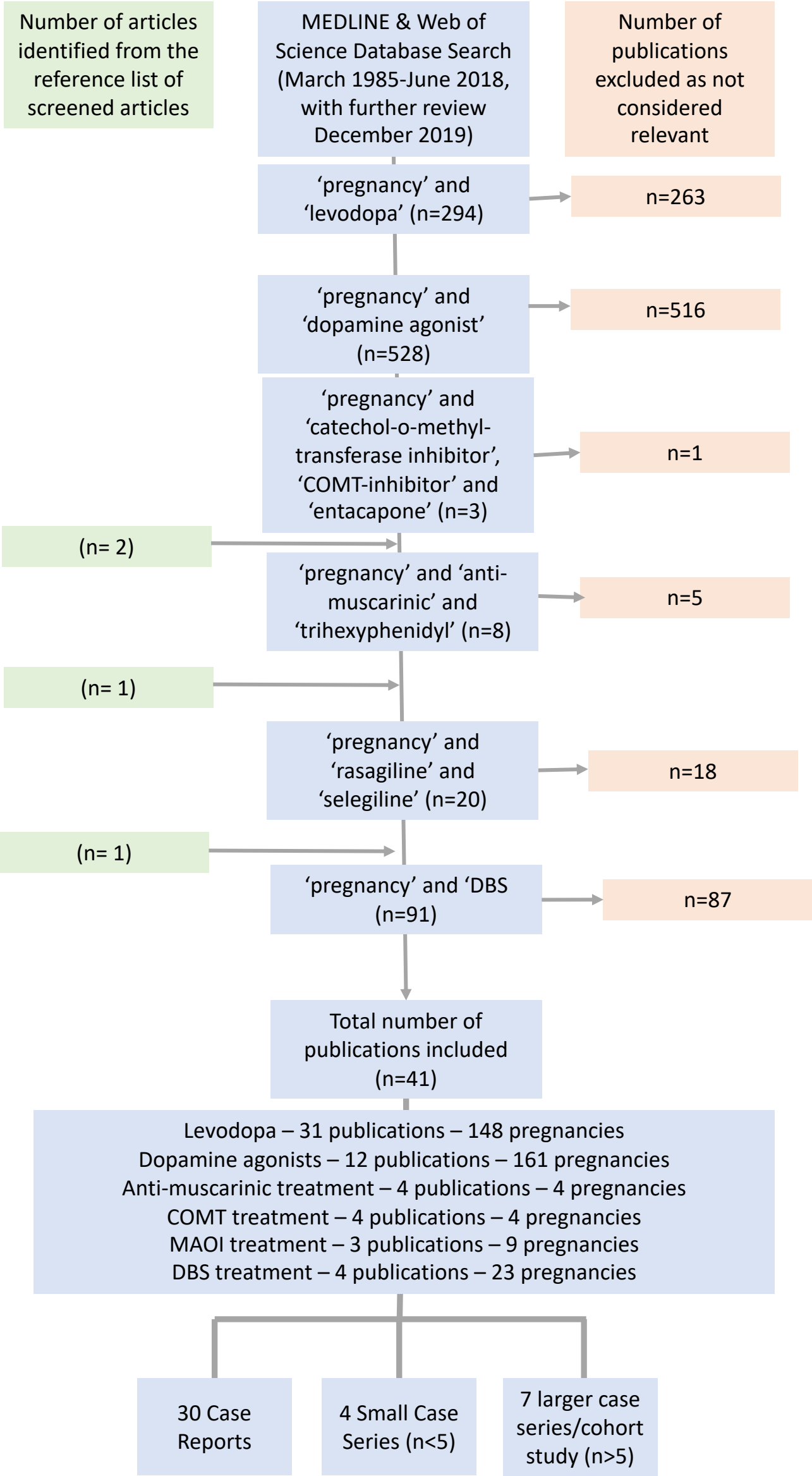

Supplement: Supplementary file 1 — Supplementary Figure S1. Schematic representation of the search terms used during the systematic literature review. Blue boxes represent the research terms used and number of publications identified. Green boxes represent additional publications identified, and orange boxes are those excluded as they were not considered relevant to this review. Articles were divided into case reports (n = 1), smaller case series (n < 5), and larger case series or cohort studies (n > 5). COMT inhibitor, catechol‐O‐methyl‐transferase inhibitor; MAOI, monoamine‐oxidase inhibitors. [file MDC3-7-419-s001.pdf]

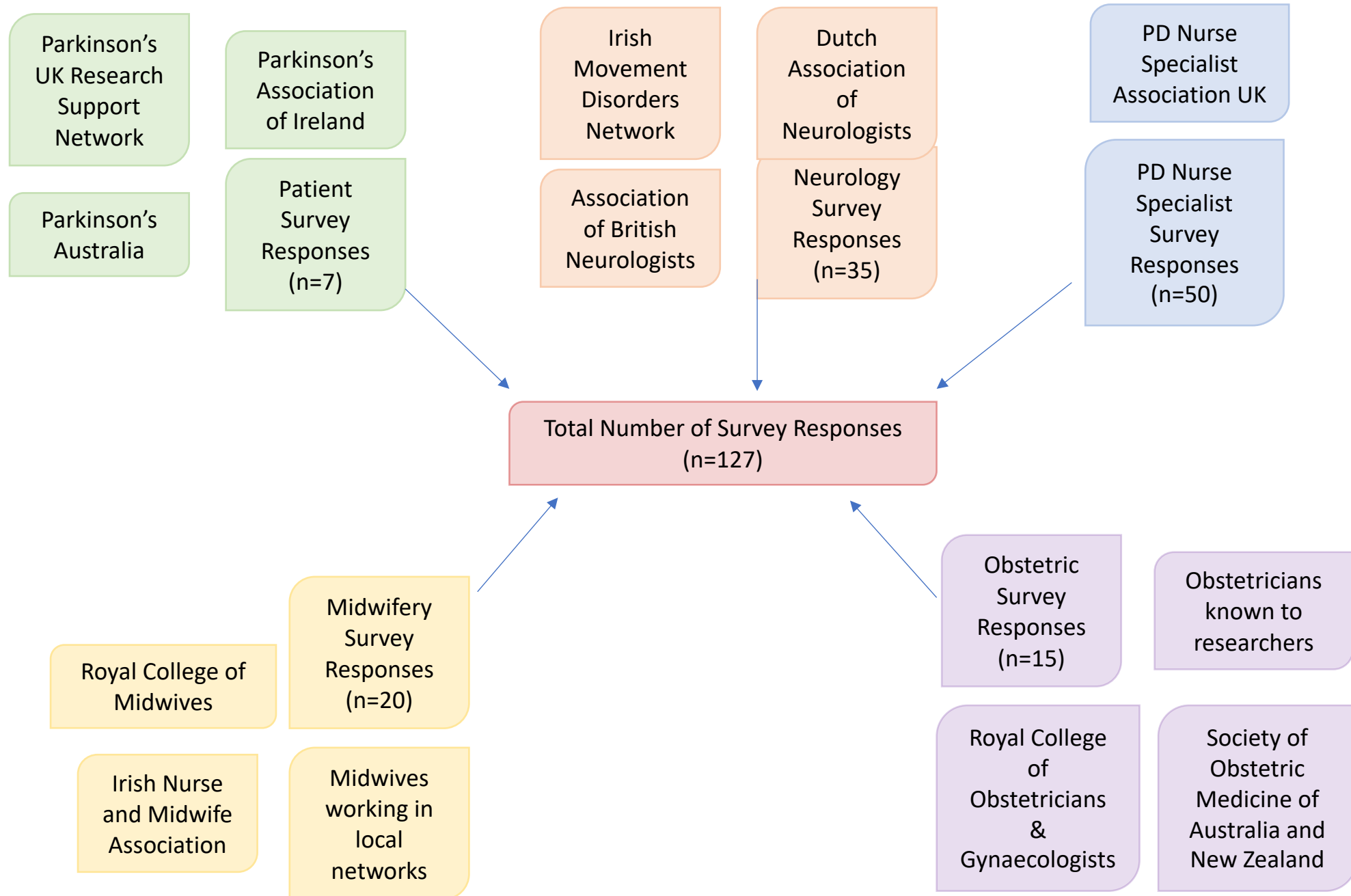

Supplement: Supplementary file 2 — Supplementary Figure S2. Schematic representation or sources of participant recruitment from clinical and patient sectors (green, patient recruitment; orange, neurology recruitment; blue, Parkinson's disease nurse specialist recruitment; yellow, midwifery recruitment; purple, obstetric recruitment). [file MDC3-7-419-s003.pdf]
