# Supplementary material for: Management of Parkinson's Disease During Pregnancy: Literature Review and Multidisciplinary Input
Source: Mov Disord Clin Pract. 2020 Apr 9;7(4):419–30. doi: 10.1002/mdc3.12925 (PMC7197310; doi:10.1002/mdc3.12925)
Supplement: Supplementary file 3 — Supplementary Table S1. Details of publications identified in systematic literature review. Supplementary Table S2. Summary of questionnaire free‐text responses. [file MDC3-7-419-s002.docx]

**Supplementary Tables**

**Supplementary Table 1: Details of publications identified in Systematic Literature Review**

| **Author** | **Year of publication** | **Cohort size – patients (pregnancies)** | **Diagnoses** | **Medications used** | **Adverse outcomes** |
| --- | --- | --- | --- | --- | --- |
| **Levodopa** | | | | | |
| Allain, H. et al. (1) | 1989 | 1(1) | PD | Levodopa |  |
| Asha, B. et al. (2) | 2010 | 1(1) | PD | Levodopa |  |
| Ball, M. C. et al. (3) | 1995 | 1(1) | P | Levodopa | AD- delayed 2^nd^ stage. No complications. |
| Basile, S. et al. (4) | 2017 | 1(1) | PD | Levodopa |  |
| Campos-Sousa, R. N. et al (5) | 2008 | 1(8) | PD | Levodopa |  |
| Cook, D. G. et al. (6) | 1985 | 2(3) | PD | Levodopa |  |
| De Mari, M. et al. (7) | 2002 | 1(1) | PD | Levodopa |  |
| Dostal, M. et al. (8) | 2013 | 42(43) | PD, RLS, SD, DRD, Suicide attempt | Levodopa | 3 SA. 7 pre-term deliveries. 3 minor anomalies (PFO+PDA, talipes varus, nasal deformity). |
| Golbe, L. I. (9) | 1987 | 5(6) | PD | Levodopa | 1 pre-eclampsia |
| Ha, D. E. et al. (10) | 2007 | 1(1) | PD | Levodopa | Pre-term delivery. PPROM at 26 and 31 weeks with EMCS at 32 weeks |
| Hagell, P. et al. (11) | 1998 | 1(1) | PD | Levodopa |  |
| Jacquemard, F. et al. (12) | 1990 | 1(1) | PD | Levodopa |  |
| Kallen, B. et al. (13) | 2013 | 37(37) |  | Levodopa |  |
| Kanzato, N. et al. (14) | 2006 | 1(1) | PD | Levodopa |  |
| Kupsch, A. et al. (15) | 1998 | 1(1) | PD | Levodopa |  |
| Lindh, J. (16) | 2007 | 1(1) | PD | Levodopa | Neonate seizure 1 hour post-partum. |
| Nomoto, M. et al. (17) | 1997 | 1(3) | DRD | Levodopa | SA at 6 and 12 weeks. |
| Nygaard, T. G. et al. (18) | 1991 | 3(3) | DRD | Levodopa |  |
| Routiot, T. et al. (19) | 2000 | 1(1) | PD | Levodopa |  |
| Scott, M. et al. (20) | 2005 | 1(2) | PD | Levodopa | Pre-term delivery by EMCS- placental abruption at 32 weeks |
| Scelzo, E. et al. (21) | 2015 | 3(6) | PD | Levodopa | 2 SA |
| Serikawa, T. et al. (22) | 2011 | 1 (1,twins) | PD | Levodopa | Pre-term delivery by EMCS- PPROM at 35 weeks. Small VSD in one twin. |
| Shulman, L. M. et al. (23) | 2000 | 1(1) | PD | Levodopa |  |
| Thulin, P. C. et al. (24) | 1998 | 1(1) | PD | Levodopa |  |
| Tüfekçioǧlu, Z. et al. (25) | 2018 | 5(5) | PD | Levodopa | 1 pre-term delivery by CS at 35 weeks, infant developed foetal distress during labour, eventually resolved. |
| von Graevenitz, K. S. et al. (26) | 1996 | 6(6) |  | Levodopa | 1 SA |
| Ward, V. D. (27) | 2018 | 1(1) | PD | Levodopa |  |
| Watanabe, T. et al. (28) | 2009 | 6(8) | SD | Levodopa | 2 SA |
| Watanabe, T. et al. (29) | 2012 | 1(1) | SD | Levodopa |  |
| Zhu, L. et al. (30) | 2011 | 1(1) | MSA | Levodopa |  |
| Zlotnik, Y. et al. (31) | 2014 | 1(1) | PD | Levodopa | Faltering growth at 13 months post-partum. |
| **Dopamine agonists** | | | | | |
| Asha, B. et al. (2) | 2010 | 1(1) | PD | ROP |  |
| Benbir, G. et al. (32) | 2014 | 1(1) | PD | PRAM | Premature delivery at 35 weeks. |
| Benito‐León, J. et al. (33) | 2001 | 1(1) | PD | BROM |  |
| De Mari, M. et al. (7) | 2002 | 1(1) | PD | PER |  |
| Dostal, M. et al. (8) | 2013 | 21(21,twins) | RLS, SD | PRAM, ROT, ROP | 3 SA, 3 premature deliveries, 1 SGA |
| Kallen, B. et al. (13) | 2013 | 117(117) | - | BROM, PRAM, APOM, CAB |  |
| Lamichhane, D. et al. (34) | 2014 | 1(1) | PD | PRAM |  |
| Lindh, J. (16) | 2007 | 1(1) | PD | BROM | Neonate seizure 1 hour post-partum |
| Mucchiut, M. (35) | 2004 | 1(1) | PD | PRAM |  |
| Scott, M. et al. (20) | 2005 | 1(2) | PD | CAB | Pre-term delivery at 32 weeks by EMCS for placental abruption. |
| Serikawa, T. et al. (22) | 2011 | 1(1, twins) | PD | ROP | Pre-term delivery by EMCS at 35 weeks for PPROM. Small VSD in one twin. |
| Tüfekçioǧlu, Z. et al. (25) | 2018 | 12(13, twins) | PD | PRAM, PIRI, ROP | 1 SA, 3 premature deliveries. Neonatal in 1 twin death due to liver enzyme deficiency.  1 prem infant developed foetal distress during labour, eventually resolved. |
| **Anti-muscarinics** | | | | | |
| Goyal, S. et al. (36) | 2017 | 1(1) | SCZ | TRI |  |
| Mendhekar, D. N. et al. (37) | 2011 | 1(3) | SCZ | TRI |  |
| Robottom, B. J. et al. (38) | 2011 | 1(2) | Dystonia | TRI |  |
| Scelzo, E. et al. (21) | 2015 | 1(1) | PD | TRI | SA |
| **COMT inhibitors** | | | | | |
| Basile, S. et al. (4) | 2017 | 1(1) | PD | ENTA |  |
| Lindh, J. (16) | 2007 | 1(1) | PD | ENTA | Neonate seizure 1 hour post-partum. |
| Serikawa, T. et al. (22) | 2011 | 1(1, twins) | PD | ENTA | Pre-term delivery by EMCS at 35 weeks due to PPROM. Small VSD in one twin. |
| Tüfekçioǧlu, Z. et al. (25) | 2018 | 1(1) | PD | ENTA |  |
| **MOA inhibitors** | | | | | |
| Kupsch, A. et al. (15) | 1998 | 1(1) | PD | SELE |  |
| Serikawa, T. et al. (22) | 2011 | 1(1, twins) | PD | SELE | Pre-term delivery by EMCS at 35 weeks due to PPROM. Small VSD in one twin. |
| Tüfekçioǧlu, Z. et al. (25) | 2018 | 7(7, twins) | PD | RASA | 2 pre-term deliveries. 1 neonatal death of a twin pregnancy due to liver enzyme deficiency. |
| **DBS during pregnancy** |  |  | **Indication** |  | **Outcome** |
| Ziman.N et al. (39)  Park H et al. (40)  Scelzo E et al. (21)  Paluzzi A et al. (41) | 2016  2017  2015  2006 | 6 (6.twins)  1(1)  11(12)  3(4) | DYSTONIA  DYSTONIA  3 PD, 5 DYSTONIA, 2 TS, 1 OCD  DYSTONIA |  | 7 live births, inc twins. 1premature delivery at 35 weeks.  Live birth by ELCS at 38 weeks.  11 live births at term; 3 VD, 9 CS. 1 SA.  4 live births |
| Legend: AD= assisted delivery, APOM= Apomorphine, BROM= bromocriptine, CAB= cabergoline, CS= caesarean section DRD= Dopa-responsive Dystonia, ELCS= elective caesarean section, EMCS= emergency caesarean section, ENTA= entacapone, HPL= hyperprolactinaemia, LTF=lost to follow-up, MSA= multiple system atrophy, OCD=obsessive compulsive disorder, P= Parkinsonism, PD= Parkinson’s disease, PDA=patent ductus arteriosus¸ PER= pergolide, PFO= patent foramen ovale, PRAM= pramipexole, PPROM= preterm premature rupture of membranes, RLS= restless leg syndrome, ROP= ropinirole, ROT= rotigotine, SA= spontaneous abortion, SCZ= schizophrenia, SD= Segawa disease, SELE= selegiline, SGA= small for gestational age, TOP= termination of pregnancy, TRI= trihexyphenidyl, TS= Tourette’s syndrome, VSD= ventricular septal defect | | | | | |
|  | | | | | |

**Supplementary Table 2: Summary of questionnaire free text responses**

| A. Neurology suggested care | |
| --- | --- |
| Medication during pregnancy | *‘I would review the potential of teratogenicity of the drugs the patient was taking and utilize the safest combination of medication for mother and child.’* ***N3***  *‘I would make sure that all therapies are optimised, to ensure Mum is as fit as possible but would balance this with lowest drug doses to achieve this’* ***N17***  *‘I would focus on treatment with L-dopa and try to minimise other drugs preferably from before conception to around 12 week.’* ***N1***  *‘I probably would continue the levodopa, and if possible, use monotherapy.’* ***N18***  *I would check with senior pharmacists, medical obstetricians and do a literature search for evidence on best practice.’* ***N8***  *‘[manage] On an individual basis, adhering to guidelines where possible’* ***N31***  *‘Consult the following sourced of information: 1) Movement Disorder Specialist, 2) Hospital Pharmacist, 3) the medical literature’* ***N29***  *‘I suspect there isn’t a great deal of data. That said, methyl dopa is used for hypertension in pregnancy and I suspect the L-dopa preparations are the safest’* ***N33*** |
| Communication | *‘Would check the safety of PD drugs on developing baby and discuss with patient.’* ***N16***  *‘Early meeting to review meds, meeting during preg to discuss delivery and post-natal care’* ***N30***  *‘Explain that pregnancy is generally safe in PD’* ***N9*** |
| Antenatal review | *‘Referral to specialist md clinic.’* ***N25***  *‘I would ensure regular PD nurse and movement disorders consultant review and liaison with Obstetrics’* ***N3***  *‘speak with their midwife and a PD CNS’* ***N26***  *‘close liaison with obstetrician and midwife.’* ***N30***  *‘I would check with senior pharmacists’* ***N8***  *‘I would seek specialist input from a Parkinson’s/movement disorders expert’* ***N35*** |
| Delivery and post-partum care | *‘Ultimately, I would want to have a plan for the birth if it looks like it may be complicated, e.g. ensuring it’s at a site with neurology cover.’* ***N26***  *‘meeting during preg to discuss delivery and post-natal care’* ***N30*** |
| B. PD Nurse Specialist suggested care |  |
| Use of medication during pregnancy | *‘Read up on medications via Electronic Medicines Compendium and local Trust guidelines/NICE best practice’* ***PDNS 37***  *‘I would gain more info re medication that can be taken during pregnancy’* ***PDNS 14***  *‘check the BNF regarding medications’* ***PDNS 27***  *‘I have no experience and with no guidelines, I would try and keep medication to a minimum.’* ***PDNS 12***  *‘Discuss with patient need for medication decide if could wean off dopaminergic therapy if taking low doses.’* ***PDNS 8***  *‘Minimise medication as much as possible. If possible, remove all but levodopa’* ***PDNS 11***  *‘Maintain stability, don't introduce any new treatments’* ***PDNS 31*** |
| Multi-disciplinary working | *‘Liaise closely with Neurologist in our service who specialises in maternal medicine and a midwife who works with him…Work with speciality midwife re symptom management.’* ***PDNS 7***  *‘In very close coordination with the patient, Consultant and GP’.* ***PDNS 36***  *‘As this is an area where I have no previous experience, I would liaise closely with the patient’s neurologist in order to develop a management plan with an agreed plan of ongoing monitoring.’* ***PDNS 15***  *‘MDT approach to include Neurologists midwives, physiotherapy. Closer monitoring’* ***PDNS 23***  *‘Having no experience, I would d be asking other PDNS for information’* ***PDNS 9***  *‘Ask local group/ PDNSA for any experience/advice.’* ***PDNS 10***  *‘I would contact colleagues who've had experience in this area and would be guided by their knowledge & experience’* ***PDNS 20***  *‘Same as epilepsy. In the same manner my colleague does with patients with epilepsy who are pregnant.’* ***PDNS 6***  *‘In close collaboration with their consultant neurologist and obstetrician.’* ***PDNS 16***  ***‘****‘MDT approach’* ***PDNS 32***  ***‘****Liaise with obstetric team.’* ***PDNS 8***  *‘I would seek advice from multi-professional team and Neurologist.’* ***PDNS 37***  *‘see if I could arrange joint working with a midwife’* ***PDNS 27***  *‘Jointly with consultant. Be available to midwife to discuss’* ***PDNS 34***  *‘Liaise with midwives.’* ***PDNS 38***  *‘Obs involvement. Positively and supportively but with caution and close involvement of the obstetrician.’* ***PDNS 2***  *‘I would look for as much information as possible e.g. on-line, journals, Parkinson's UK, other Parkinson's Nurses, on-line forum on the members area of Parkinson's Disease Nurse Specialist Association website, the patient's Parkinson's Consultant and Consultant Obstetrician, pharmacists.’* ***PDNS 3***  *‘liaise with maternity team’* ***PDNS18***  *‘Liaise with the obstetricians.’* ***PDNS 30*** |
| Antenatal review | *‘Emphasise their open access to the service.’* ***PDNS 10***  *‘Increase visits’* ***PDNS 18***  *‘Closer monitoring’* ***PDNS 23***  *‘Frequent reviews’. ‘Ask patient to keep symptoms diary’* ***PDNS 34*** |
| C. Obstetrics suggested care |  |
| Reasons for obstetric-led antenatal review | *‘with medication involved’* ***O6***  *‘would want to ensure no decline during pregnancy’* ***O7***  *‘given the unknown challenges and the need for a multidisciplinary approach’* ***O4***  *‘Multidisciplinary team including a medical midwife, obstetric physician and MFM obstetrician with advice from the patients primary neurologist’* ***O12***  *‘extra reassurance, fetal growth scans, MDT input with close liaison between Obs, neuro and anaesthetics, consideration of VTE prophylaxis etc’* ***O10***  *‘Could be either - if early in disease process and no concerns, MLC might be best. CLC would ensure liaison with neurology colleagues, contact with nurse specialist and joint planning for delivery and postnatal’* ***O14***  *‘would be easier to arrange follow-ups and implement any management plan’* ***O15*** |
| Schedule of antenatal care | *‘as standard regimen, more frequently if progressive Parkinson’s disease activity’* ***O4***  *‘standard obstetric care’* ***O6***  *‘care as usual and more often based on complaints’* ***O5***  *‘Pre-pregnancy counselling’* ***O12***  *‘[appointments] Ideally coinciding with fetal growth scans.’* ***O10***  *‘Regular monthly joint clinic’* ***O2*** |
| Frequency of discussion with neurology team | *‘Regular monthly joint clinic and contact if any concerns’* ***O2***  *‘Once or twice if no problems, more frequently if progressive Parkinson’s disease activity’* ***O4***  *‘Once in the beginning of pregnancy and in the third trimester, also with anaesthetist’* ***O6***  *‘At least each trimester.’* ***O10***  *‘General planning discussion preferably pre-pregnancy, otherwise when booking with our clinic. Discussion with neurology after that only if needed or if altering medications’* ***O12***  *‘Pre-conception .At booking . With change’* ***O13***  *‘At booking, following any significant clinical change or change of medication, prior to delivery for planning’* ***O15*** |
| Suggested delivery | *‘Not [c-section] unless there were other obstetric indications’* ***O12***  *‘No [c-section]. I would reserve section for Obstetric reasons only. There are pros and cons of this, but, as with many medical Obs patients, we would encourage an early epidural, offer induction of labour at term (to give some ‘predictability’) and have an honest dialogue with the pt and whole MDT about the birth plan.’* ***O10***  *‘Not routinely - there can be allowances made to minimise active (pushing) phase’* ***O15*** |
| Post-partum care | *‘Depending of level of disability likely to need longer postnatal hospital stay and occupational therapy input around infant cares’* ***O12***  *‘Monitor patient in HDU on the delivery suite for 24 hours postpartum. Be aware of potential for worsening on symptoms. Neurology review within 24 hours of delivery.’* ***O10***  *‘ensure support from midwives’* ***O7***  *‘Neurology follow up’* ***O14*** |
| D. Midwifery suggested care |  |
| Antenatal review | *‘Obstetric referral, Obstetric led care’* ***M2***  *‘During pregnancy I would expect the woman to be placed under consultant led care with multi-disciplinary input…The woman would also see her midwife in community to provide normal antenatal care, monitoring, and emotional support throughout the pregnancy.’ ‘Physiotherapy input to reduce the risk of falls and therefore injury to the woman and fetus.’ ‘There may also be serial ultrasound scans to monitor fetal growth and development.’* ***M3***  *‘I would mainly think about how well she is able to access antenatal care, possible home midwife visits maybe more appropriate than possible long waits in clinics.’ ‘Perhaps seeing a physio would help with balance issues (also antenatally).’* ***M1***  *‘liaise with other health professional involved in current care. consultant led. foetal medicine early pregnancy. care physio referral. anaesthetic referral. Perni mental health team. assess if able to breast’* ***M14***  *‘Booking and antenatal care: refer to consultant led care, refer for a specialist nurse who deals with Parkinson’s , more antenatal appointment due to ?higher risk in pregnancy.’* ***M15***  *‘assess mobility. consider serial growth scans due to medication’* ***M16***  *‘that the woman is seen by her medical, obstetric & anaesthetic team to ensure that a multi disciplinary care plan for her antenatal care & her delivery are made. the lady should be involved in all aspects of her care & the normal care pathway should be followed as much as possible.’* ***M19*** |
| Intrapartum care | *‘In labour, my train of thought is that she might tire easily if her Parkinson’s is severe, so advising her periods of rest as well as helping her to be somewhat mobile during labour.’*  ***M1***  *‘During labour care and the woman might have regular mobility checks to reduce the risk of falls whilst also recommending active labour where possible.’* ***M3***  *‘physical support in labour’* ***M5***  *‘Labour care’* ***M10***  *‘delivery in an Obstetric unit or Midwifery led unit alongside an Obstetric unit.’* ***M2***  *‘The obstetricians would decide whether an early induction of labour 37-39 weeks gestation would be of benefit to ensure the best outcome.’* ***M3*** |
| Post-partum care | *‘Postnatal period I’d like so support her with how she handles her baby and how she can manage this with her movements.’* ***M1***  *‘I also think it would be a good idea during pregnancy for the woman to have advise on caring for a newborn with her condition.’* ***M3***  *‘assessment of adapted care for baby following delivery’* ***M5***  *‘postnatal care afterwards’* ***M8*** |

**References**

1. Allain H, Bentue-Ferrer D, Milon D, et al. Pregnancy and Parkinsonism: A Case Report Without Problem. *Clin Neuropharmacol* 1989;12(3):217-9.

2. Asha B, Hansali N, Apoorva P. Successful birth of an IVF baby in a patient with Parkinson's disease. *J Hum Reprod Sci* 2010;3(1):42-3.

3. Ball MC, Sagar HJ. Levodopa in pregnancy. *Mov Disord* 1995;10(1):115.

4. Basile S, Pinelli S, Garibaldi S, et al. Cathecol-O-methyltransferase inhibitors: another possibly useful pharmacological tool for treating Parkinson's disease in pregnancy? *J Obstet Gynaecol* 2017;37(3):381-2.

5. Campos-Sousa RN, Almeida KJ, Dos Santos AR, et al. Multiparity after an initial diagnosis of Parkinson's disease: a report on a rare case. *Fertil Steril* 2008;90(5):2005.e1-2.

6. Cook DG, Klawans HL. Levodopa during pregnancy. *Clin Neuropharmacol* 1985;8(1):93-5.

7. De Mari M, Zenzola A, Lamberti P. Antiparkinsonian treatment in pregnancy. *Mov Disord* 2002;428-9.

8. Dostal M, Weber-Schoendorfer C, Sobesky J, et al. Pregnancy outcome following use of levodopa, pramipexole, ropinirole, and rotigotine for restless legs syndrome during pregnancy: a case series. *Eur J Neurol* 2013;20(9):1241-6.

9. Golbe LI. Parkinson's disease and pregnancy. *Neurology* 1987;37(7):1245-9.

10. Ha DE, Legendre G, Colau JC. Maladie de Parkinson juvénile et prématurité récidivante. À propos d'un cas. *Gynecol Obstet Fertil* 2007;35(3):224-7.

11. Hagell P, Odin P, Vinge E. Pregnancy in Parkinson's disease: a review of the literature and a case report. *Mov Disord* 1998;13(1):34-8.

12. Jacquemard F, Palaric JC, Allain H, et al. Parkinson disease and pregnancy. Apropos of a case. *J Gynecol Obstet Biol Reprod* 1990;19(4):461-3.

13. Kallen B, Borg N, Reis M. The use of central nervous system active drugs during pregnancy. *Pharmaceuticals (Basel)* 2013;6(10):1221-86.

14. Kanzato N, Nishihira T, Murao H, at al. Case of juvenile parkinsonism in pregnancy. *Rinsho Shinkeigaku* 2006;46(6):400-3.

15. Kupsch A, Oertel WH. Selegiline, pregnancy, and Parkinson's disease. *Mov Disord* 1998;13(1):175-6.

16. Lindh J. Short episode of seizures in a newborn of a mother treated with levodopa/carbidopa/entacapone and bromocriptine. *Mov Disord* 2007;22(10):1515.

17. Nomoto M, Kaseda S, Iwata S, et al. Levodopa in pregnancy. *Mov Disord* 1997;12(2):261.

18. Nygaard TG, Marsden CD, Fahn S. Dopa-responsive dystonia: long-term treatment response and prognosis. *Neurology* 1991;41(2 ( Pt 1)):174-81.

19. Routiot T, Lurel S, Denis E, et al. Parkinson's disease and pregnancy: case report and literature review. *J Gynecol Obstet Biol Reprod*. 2000;29(5):454-7.

20. Scott M, Chowdhury M. Pregnancy in Parkinson's disease: unique case report and review of the literature. *Mov Disord* 2005;20(8):1078-9.

21. Scelzo E, Mehrkens JH, Bötzel K, et al. Deep Brain Stimulation during Pregnancy and Delivery: Experience from a Series of “DBS Babies”. *Front Neurol* 2015;6:191.

22. Serikawa T, Shimohata T, Akashi M, et al. Successful twin pregnancy in a patient with parkin-associated autosomal recessive juvenile parkinsonism. *BMC Neurol*. 2011;11:72.

23. Shulman LM, Minagar A, Weiner WJ. The effect of pregnancy in Parkinson's disease. *Mov Disord* 2000;15(1):132-5.

24. Thulin PC, Woodward WR, Carter JH, et al. Levodopa in human breast milk Clinical implications. *Neurology* 1998;50(6):1920.

25. Tüfekçioğlu Z, Hanağası H, Yalçın Çakmaklı G, et al. Use of anti-Parkinson medication during pregnancy: a case series. *J Neurol* 2018;265(8):1922-9.

26. von Graevenitz KS, Shulman LM, Revell SP. Levodopa in pregnancy. *Mov Disord* 1996;11(1):115-6.

27. Ward VD. Anaesthesia for Caesarean section in a patient with Parkinson's disease. *Int J Obstet Anesth* 2018;34:99-102.

28. Watanabe T, Matsubara S, Baba Y, et al. Successful management of pregnancy in a patient with Segawa disease: case report and literature review. *J Obstet Gynaecol Res* 2009;35(3):562-4.

29. Watanabe T, Matsubara S. Good obstetric outcome in a patient with Segawa disease. *Arq Neuropsiquiatr* 2012;70(7):559-60.

30. Zhu L, Cairns NJ, Tabbal SD, et al. Pregnancy in multiple system atrophy: a case report. *J Med Case Rep* 2011;5:599-.

31. Zlotnik Y, Giladi N, Hilel A, et al. Levodopa-carbidopa intestinal gel (LCIG) infusion during pregnancy and delivery: first documented case. *Parkinsonism Relat Disord* 2014;20(11):1317-8.

32. Benbir G, Ertan S, Ozekmekci S. Successful pregnancy and delivery in a patient with Parkinson's disease under pramipexole treatment. *Presse Med* 2014;43(1):83-5.

33. Benito‐León J, Bermejo F, Porta‐Etessam J. Pregnancy in Parkinson's disease: A review of the literature and a case report. *Mov Disord* 2001;14(1):194-.

34. Lamichhane D, Narayanan NS, Gonzalez-Alegre P. Two cases of pregnancy in Parkinson’s disease. *Parkinsonism Relat Disord* 2014;20(2):239-40.

35. Mucchiut M. Pramipexole-treated Parkinson's disease during pregnancy. *Mov Disord* 2004;19:1114-5.

36. Goyal SK, Goel A. Successful Use of Risperidone, Trihexyphenidyl, and Paroxetine in Pregnancy. *Indian J Psychol Med* 2017;39(6):835-6.

37. Mendhekar DN, Andrade C. Uneventful use of haloperidol and trihehexyphenidyl during three consecutive pregnancies. *Arch Womens Ment Health* 2011;14(1):83-4.

38. Robottom BJ, Reich SG. Exposure to high dosage trihexyphenidyl during pregnancy for treatment of generalized dystonia: case report and literature review. *Neurologist* 2011;17(6):340-1.

39. Ziman N, Coleman R, R, Starr P, A, Volz M, Marks Jr. W, J, Walker H, C, Guthrie S, L, Ostrem J, L: Pregnancy in a Series of Dystonia Patients Treated with Deep Brain Stimulation: Outcomes and Management Recommendations. Stereotact Funct Neurosurg 2016;94:60-65. doi: 10.1159/000444266

40. Park HR, Lee JM, Park H, Shin CW, Kim HJ, Park HP, Kim DG, Jeon BS, Paek SH: Pregnancy and Delivery in Generalised Dystonia Patient Treated with Internal Globus Pallidal Deep Brain Stimulation: A Case Report.J Korean Med Sci 2017; Jan;32(1):155-159. doi: 10.3346/jkms.2017.32.1.155

41. Paluzzi A, Bain PG, Liu X, Yianni J, Kumarendran K, Aziz TZ: Pregnancy in dystonic women with in situ deep brain stimulators. Mov Disord 2006 May;21(5):695-8.
